# Supplementary material for: Self /other recognition and distinction in adolescents with anorexia nervosa: A pilot study using a double mirror paradigm
Source: PLoS One. 2025 Jan 2;20(1):e0309548. doi: 10.1371/journal.pone.0309548 (PMC11695000; doi:10.1371/journal.pone.0309548)
Supplement: S1 Raw data — (PDF) [file pone.0309548.s001.pdf]

	M1Neu	M2Neu	M1RHB	M2RHB	M1LHB	M2LHB	M1BFR
A1	70	150	110	150	130	140	80
B1	130	160	120	120	130	130	140
A2	90	140	80	110	80	120	150
B2	130	140	140	150	130	140	100
A3	110	150	120	130	120	120	140
B3	150	140	160	140	140	140	140
A4	60	120	80	110	60	110	130
B4	120	160	140	150	140	150	130
A5	70	130	70	120	70	120	70
B5	160	160	170	150	140	150	150
A6	80	130	70	110	70	130	90
B6	130	140	150	130	140	150	140
A7	60	130	60	90	80	90	90
B7	110	150	80	150	80	130	80

M2BFR	M1UC	M2UC	M1NP	M2NP
130	80	110	90	130
130	130	130	150	130
150	100	130	100	110
140	140	140	110	140
120	100	110	100	110
140	160	150	150	130
90	60	110	70	100
110	140	140	130	150
120	50	100	60	100
150	160	160	140	150
130	100	100	90	120
130	140	130	140	130
130	70	120	80	110
100	80	130	90	120
