# Supplementary material for: Self /other recognition and distinction in adolescents with anorexia nervosa: A pilot study using a double mirror paradigm
Source: PLoS One. 2025 Jan 2;20(1):e0309548. doi: 10.1371/journal.pone.0309548 (PMC11695000; doi:10.1371/journal.pone.0309548)
Supplement: Attachment — Submitted filename: Review PLOS ONE manuscript 2023.docx [file pone.0309548.s002.docx]

**Review PLOS ONE manuscript (PONE-D-23-07323) July 2023**

**Summary.** The aim of this pilot study was to examine self-recognition and self/other distinction (SOD) in seven adolescents with anorexia nervosa (AN) and in seven matching controls (HC) by using a self-versus other face identification task through a double mirror ”Alter Ego”TM device (developed by Moritz Wehrmann) that provide an ability “*to progressively change the identity of self and other in the mirror with changes in lighting on both sides of the device*” (p. 7).

The authors presents previous and relevant research on body image and body schema issues, and discuss limitations associated with dysfunctional beliefs and cognitions associated with body image, and lack of reference to social processes involved in the relations between self and others in research focusing on the representation of the physical body and body schema issues. The authors then offer a reasoning for addressing self/other distinction in individuals with AN. Self/other distinction and the ability to “*differentiate one´s own body, actions, and mental states from those of others is crucial for establishing relationships with others while maintaining a stable sense of self*” (p.4). In lacking such capacity, confusion between self and other may occur. Previous research on SOD in the general population and in patients with mental disorders (including AN) have mostly used static images or movies, and are considered insufficient for SOD assessment. It is argued that to better understand the underpinnings of AN, studies should take on *an embodied perspective* involving participants who are physically present.

Criteria for inclusion and exclusion of participants and controls are well described. Descriptions of the experiment set-up, and the two different procedures, the neutral condition and the sensorimotor condition, are sound and convincing. The two judgement criteria used were: M1, the threshold at which subjects start to recognize their own face during other-to-self morphing, and M2, the threshold at which subjects start to recognize the other´s face during self-to-other morphing. A specialist in statistics has assisted with the statistical analysis.

The results from *neutral condition* showed that earlier self-recognition in the other-to-self and delayed other-recognition in the self-to-other sequence in participants with AN compared to the HC. As opposed to that of controls, the critical threshold for switching between self and other varied with the direction of morphing in participants with AN.

The main finding measured during the *sensorimotor condition*, when the subjects with AN were seated in a chair where they were touching the back- and footrest (BFR) and receiving some postural support, the self-recognition threshold (M1) increased significantly and was nearer to that of controls. In this position, the AN subjects showed delayed self-recognition as they continued to perceive the other longer while their own image was gradually appearing. The findings and their implications are thoroughly discussed in relation to other research, and it is theorized whether the use of postural support might have affected self-recognition and SOD in subjects with AN.

The focus of the paper is recognition of self and other in individuals with AN. The results suggest that disturbances in mirror self-recognition indicates bodily self-consciousness impairments in subjects with AN, that subjects with AN are more egocentric in SOD compared to HC´s, and that self-recognition and SOD thresholds change when postural conditions in subjects with AN are modified. The theme of the paper is important and the study contributes to increased understanding of SOD in individuals with AN. It is well written and it refer to a number work relating to SOD, while some theoretical and conceptual aspects needs some further elaboration. Having done that, the article would be recommended for publication.

**Concerns**

There are two concerns related to the theoretical and conceptual foundations of the study:

1. What about the self in individuals with AN? In the presentation of the rationale behind exploring self/other distinction in individuals with AN, the authors claim that a stable sense of self is crucial in order to relate to the social world and establish relationships with others, as well as for differentiating one´s own body from those of others (p. 4). It could, however, also be argued that to be able to differentiate oneself from the other, there must be a stable sense of self. But what is it that constitutes a “stable sense of self” in this context? Having a psychiatric disorder involve transformations to one´s sense of self and one´s body, and the way one inhabits, or live, one´s body is changed individuals with AN. See for example Legrand (1), which you already have referenced, who claims that people with AN often lack the experience of the body as an integrated place of their own. How can these ideas affect the ability to accurately recognize others?

2. The second concern is about the concept of embodiment and whether “involving participants who are physically present” is equivalent to an embodied perspective.

The embodiment thesis infers that the subject (and perception of the self) is constitutively bodily (2, p 208). To be an experiencing subject, one needs to be an experiencing body as well. Even though the subjects were physically present in this study, were they also embodied, in the sense that the body is the source of subjectivity, feelings, perceptions and sensations? Given that only the participants head and faces were visual in the experiments, did this speak more to visual recognition and cognition than proprioception and embodiment? As the results of the study show, when providing the subjects with backrest and footrest, and the possibility of receiving bodily support from these, bodily tension (AN is strongly associated with high levels of anxiety, which tend to produce postural stiffness, muscular tension, and constricted breathing) might have lessened and contributed to the subjects being more present in the here and now. The authors of a recent article on the lived body, found that in allowing themselves to take support from the seat and back of the chair (as part of physiotherapy consultations, which also involved other means), instead of perching on the edge of the chair, subjects with AN felt they gained a feeling of becoming more attentive and of being more present in the here and now (3). Given that embodiment, perception, action and subjectivity are inseparable, such changes to the body may thus invoke changes in one´s sense of self and one´s way of being in the world.

Other points

This reviewer have limited competence in statistics, but have confidence in the work done by the specialist and that the statistics will be considered by other reviewers.

A few spelling issues:

p 11, line 13: strategies (lack r),

p 14, second line in section on “Neutral condition”: “individuals with AN” x 2?

**References**

1.	Legrand D. Subjective and physical dimensions of bodily self-consciousness, and their dis-integration in anorexia nervosa. Neuropsychologia. 2010;48:726-37.

2.	Legrand D. Phenomenological dimensions of bodily self-consciousness. In: Gallagher S, editor. The Oxford Handbook of the Self. New York: Oxford university press; 2011. p. 204-27.

3.	Naess CR, Kolnes L-J. A preliminary exploration of experiences of integrating the body in the self in two women with anorexia nervosa in view of phenomenological conceptualisations. Journal of eating disorders. 2022;10.
