# Supplementary material for: Self /other recognition and distinction in adolescents with anorexia nervosa: A pilot study using a double mirror paradigm
Source: PLoS One. 2025 Jan 2;20(1):e0309548. doi: 10.1371/journal.pone.0309548 (PMC11695000; doi:10.1371/journal.pone.0309548)
Supplement: Attachment — Submitted filename: Revision PONE-D-23-07323.docx [file pone.0309548.s003.docx]

Thank you for giving me the opportunity to review this interesting study. In my opinion, the study is very innovative and original. However, I have some concerns about the current manuscript.

*Abstract*

- The abstract could be improved
- Anorexia Nervosa should be written with upper cases
- I suggest to use the expression “Participants affected by AN” instead of “with AN”

*Introduction*

- It is not clear the link between body distortion and self-others impairments (pag. 2/3) that lead authors to their research question. Maybe this relationship might be better explained to the reader.

*Methods*

- The sample size is very small. We suggest to recruit participants, in order to have at least 15 participants for group. Because of the interesting topic and the novelty of the methology, this would improve the value of this manuscript.
- Moreover, no pathological features are reported (subtype of diagnosis, current pharmacological treatment).
- I suggest to include other measures based on literature: for instance, body distortion might be associated with altered SOD.
- I would evaluate also visual abilities across groups (use of lens, glasses, other visual-corrective devices)

*Discussion*

- Your results might be also related to multisensory integration deficits as highlighted by a recent systematic review. I suggest to include this aspect in the discussion section (for instance. Brizzi et al., 2023).
- I suggest to extend the part related to egocentric-allocentric spatial frames (pag. 16-17)
- *“In particular, individuals with AN have been found to exhibit greater sensitivity to the rubber hand illusion (Botvinick & Cohen, 1998), indicative of a greater susceptibility to appropriating and embodying an object external to their own body, i.e., a more plastic bodily-self (Eshkevari et al., 2012)*” (pag. 18) – Actually, there are contrasted results concerning this aspect so I suggest a deeper discussion about contrasting results (for instance, Provenzano et al., 2019)
- “*These results may indicate that, in the AN individuals, the proprioceptive system would be less vulnerable to bias originating from visual information (i.e., AN individuals depend more heavily on proprioceptive information than on visual information when incongruent)*.” (pag. 19) – I would appreciate some evidence related to this aspect, since a recent systematic review highlithed interoceptive deficits in AN (see Malighetti et al., 2022)

Limitations

- What are other domains of SOD (pag. 23)?
- “*in the second part of the study, the postural and sensorimotor tasks were only applied to participants with AN. Therefore, the effects of these tasks in healthy subjects remained unknown*” (pag. 23) – I do not understand the reasons for this choice from a mythological point of view.

In light of these considerations, I suggest authors to collect more data first of all. This would also improve the analysis. All other changes are minors and more related to a deeper analysis of the literature and/or style.
