# Supplementary material for: Self /other recognition and distinction in adolescents with anorexia nervosa: A pilot study using a double mirror paradigm
Source: PLoS One. 2025 Jan 2;20(1):e0309548. doi: 10.1371/journal.pone.0309548 (PMC11695000; doi:10.1371/journal.pone.0309548)
Supplement: Attachment — Submitted filename: Response to Reviewers.docx [file pone.0309548.s004.docx]

**RESPONSE TO REVIEWERS**

**REVIEWER 1**

**Summary.** The aim of this pilot study was to examine self-recognition and self/other distinction (SOD) in seven adolescents with anorexia nervosa (AN) and in seven matching controls (HC) by using a self-versus other face identification task through a double mirror ”Alter Ego”TM device (developed by Moritz Wehrmann) that provide an ability “*to progressively change the identity of self and other in the mirror with changes in lighting on both sides of the device*” (p. 7).

The authors presents previous and relevant research on body image and body schema issues, and discuss limitations associated with dysfunctional beliefs and cognitions associated with body image, and lack of reference to social processes involved in the relations between self and others in research focusing on the representation of the physical body and body schema issues. The authors then offer a reasoning for addressing self/other distinction in individuals with AN. Self/other distinction and the ability to “*differentiate one´s own body, actions, and mental states from those of others is crucial for establishing relationships with others while maintaining a stable sense of self*” (p.4). In lacking such capacity, confusion between self and other may occur. Previous research on SOD in the general population and in patients with mental disorders (including AN) have mostly used static images or movies, and are considered insufficient for SOD assessment. It is argued that to better understand the underpinnings of AN, studies should take on *an embodied perspective* involving participants who are physically present.

Criteria for inclusion and exclusion of participants and controls are well described. Descriptions of the experiment set-up, and the two different procedures, the neutral condition and the sensorimotor condition, are sound and convincing. The two judgement criteria used were: M1, the threshold at which subjects start to recognize their own face during other-to-self morphing, and M2, the threshold at which subjects start to recognize the other´s face during self-to-other morphing. A specialist in statistics has assisted with the statistical analysis.

The results from *neutral condition* showed that earlier self-recognition in the other-to-self and delayed other-recognition in the self-to-other sequence in participants with AN compared to the HC. As opposed to that of controls, the critical threshold for switching between self and other varied with the direction of morphing in participants with AN.

The main finding measured during the *sensorimotor condition*, when the subjects with AN were seated in a chair where they were touching the back- and footrest (BFR) and receiving some postural support, the self-recognition threshold (M1) increased significantly and was nearer to that of controls. In this position, the AN subjects showed delayed self-recognition as they continued to perceive the other longer while their own image was gradually appearing. The findings and their implications are thoroughly discussed in relation to other research, and it is theorized whether the use of postural support might have affected self-recognition and SOD in subjects with AN.

The focus of the paper is recognition of self and other in individuals with AN. The results suggest that disturbances in mirror self-recognition indicates bodily self-consciousness impairments in subjects with AN, that subjects with AN are more egocentric in SOD compared to HC´s, and that self-recognition and SOD thresholds change when postural conditions in subjects with AN are modified. The theme of the paper is important and the study contributes to increased understanding of SOD in individuals with AN. It is well written and it refer to a number work relating to SOD, while some theoretical and conceptual aspects needs some further elaboration. Having done that, the article would be recommended for publication.

**Concerns**

There are two concerns related to the theoretical and conceptual foundations of the study:

1. What about the self in individuals with AN? In the presentation of the rationale behind exploring self/other distinction in individuals with AN, the authors claim that a stable sense of self is crucial in order to relate to the social world and establish relationships with others, as well as for differentiating one´s own body from those of others (p. 4). It could, however, also be argued that to be able to differentiate oneself from the other, there must be a stable sense of self. But what is it that constitutes a “stable sense of self” in this context? Having a psychiatric disorder involve transformations to one´s sense of self and one´s body, and the way one inhabits, or live, one´s body is changed individuals with AN. See for example Legrand (1), which you already have referenced, who claims that people with AN often lack the experience of the body as an integrated place of their own. How can these ideas affect the ability to accurately recognize others?

First of all, the authors would like to thank the reviewer for his very attentive proofreading and pertinent comments, particularly with regard to his knowledge of the phenomenological approach to anorexia, which fits in perfectly with our approach using the double mirror in this disorder.

Indeed, certain theoretical and conceptual aspects deserved to be developed, in particular by emphasizing the phenomenological approach to anorexia, which allows access to an integrative and multidimensional conception of bodily self-consciousness that does not leave out the interpersonal and intersubjective aspects of the disorder.

This has enabled us to address the problem of objectification of the body and the tension between the first-person lived body and the third-person object body, as well as to introduce more clearly the question of the relevance of anomalies in the boundaries between self/other in anorexia nervosa within the introduction section.

2. The second concern is about the concept of embodiment and whether “involving participants who are physically present” is equivalent to an embodied perspective.

The embodiment thesis infers that the subject (and perception of the self) is constitutively bodily (2, p 208). To be an experiencing subject, one needs to be an experiencing body as well. Even though the subjects were physically present in this study, were they also embodied, in the sense that the body is the source of subjectivity, feelings, perceptions and sensations? Given that only the participants head and faces were visual in the experiments, did this speak more to visual recognition and cognition than proprioception and embodiment?

Given that only the participants' heads and faces were visualized in our experiment, the author of the article raises the question of whether our study is more about visual recognition and cognition than proprioception and embodiment.

Firstly, to take account of this pertinent comment, we have added a paragraph highlighting the special status of recognizing one's own face in the mirror from a phenomenological perspective as follows:

« From a phenomenological perspective, the image reflected in the mirror also reveals the ambiguity of realizing one's body as an object visible from the outside or by others, and yet being that object oneself - in other words, a tension between a primary embodied first person perspective and an internalized third-person perspective (Mancini, 2021). This intrapersonal tension also becomes an interpersonal one : while realizing that I am objectified or even reified by the gaze of the other, I must resist this gaze and (re)assert my own subjectivity, even if it is by objectifying the other in turn (Sartre, 2018). »

Furthermore, from a neuroscientific perspective, several studies have also provided important insights into how face identification is linked not only to visual or cognitive skills, but also to the multisensory integration of body-related signals in healthy volunteers (Tajadura-Jiménez et al., 2012). Indeed, it has been shown that face identification integrates not only visual information, but also kinesthetic, proprioceptive, visceroceptive and tactile, visual and tactile data, similar to bodily self-consciousness (Tsakiris, 2008; Sforza et al., 2009; Paladino et al., 2010; Apps et al..., 2015). Furthermore, using the double-mirror paradigm, Thirioux et al. demonstrated bidirectional links between face recognition in the mirror and bodily self-consciousness by showing that changes in self-face identification induce a bottom-up shift between the current visual representation of one's own face and the memorized representation of one's own face, which in turn has a top-down impact on bodily self-consciousness.

As the results of the study show, when providing the subjects with backrest and footrest, and the possibility of receiving bodily support from these, bodily tension (AN is strongly associated with high levels of anxiety, which tend to produce postural stiffness, muscular tension, and constricted breathing) might have lessened and contributed to the subjects being more present in the here and now. The authors of a recent article on the lived body, found that in allowing themselves to take support from the seat and back of the chair (as part of physiotherapy consultations, which also involved other means), instead of perching on the edge of the chair, subjects with AN felt they gained a feeling of becoming more attentive and of being more present in the here and now (3). Given that embodiment, perception, action and subjectivity are inseparable, such changes to the body may thus invoke changes in one´s sense of self and one´s way of being in the world.

A small number of studies exist in which the aim was to enhance body awareness (often referred to as ‘body awareness therapies’) and to apply body-oriented interventions for subjects with AN.

The authors would like to thank the reviewer for his in-depth knowledge of the literature, and this bibliographic recommendation, which provides useful support for their findings. The results of this study have been added to the discussion section.

Other points

This reviewer have limited competence in statistics, but have confidence in the work done by the specialist and that the statistics will be considered by other reviewers.

The authors thank the reviewer for his confidence

A few spelling issues:

p 11, line 13: strategies (lack r),

p 14, second line in section on “Neutral condition”: “individuals with AN” x 2?

The authors thank the reviewer for his vigilance. Corrections have been made to the manuscript

**References**

1.	Legrand D. Subjective and physical dimensions of bodily self-consciousness, and their dis-integration in anorexia nervosa. Neuropsychologia. 2010;48:726-37.

2.	Legrand D. Phenomenological dimensions of bodily self-consciousness. In: Gallagher S, editor. The Oxford Handbook of the Self. New York: Oxford university press; 2011. p. 204-27.

3.	Naess CR, Kolnes L-J. A preliminary exploration of experiences of integrating the body in the self in two women with anorexia nervosa in view of phenomenological conceptualisations. Journal of eating disorders. 2022;10.

The authors would like to thank the reviewer for these suggestions, which helped to complete their bibliography.

**Reviewer 2**

Thank you for giving me the opportunity to review this interesting study. In my opinion, the study is very innovative and original. However, I have some concerns about the current manuscript.

The authors would like to thank the reviewer most sincerely for his careful proofreading and his well-informed comments on a very specific and original topic.

*Abstract*

- The abstract could be improved
- Anorexia Nervosa should be written with upper cases
- I suggest to use the expression “Participants affected by AN” instead of “with AN”

The authors would like to thank the reviewer for his comments:

The abstract has been improved: Anorexia nervosa was written with upper cases and the expression "Participants affected by anorexia nervosa" has replaced "with AN".

*Introduction*

- It is not clear the link between body distortion and self-others impairments (pag. 2/3) that lead authors to their research question. Maybe this relationship might be better explained to the reader.

The authors would like to thank the reviewer for this request for clarification regarding the relationship between body distortion abnormalities in anorexia nervosa and self-others impairments.

In particular, this relationship is based on an abundant literature that emphasizes the central role of the body in establishing a clear distinction between self and other (Paladino et al., 2010, Tajadura-Jiménez A et al. 2012, Tsakiris et al., 2017, Palmer &Tsakiris, 2018).Especially, it is bodily self-consciousness based on multisensory integration processes that underpins the distinction between self and other (Keromnes et al., 2019). The presentation of multisensory integration anomalies previously described in AN therefore raises the question of possible anomalies in the distinction between self and other.

This transition between body distortion abnormalities and SOD impairments has been made more explicit for the reader by the addition of an extra paragraph within the introduction

*Methods*

- The sample size is very small. We suggest to recruit participants, in order to have at least 15 participants for group. Because of the interesting topic and the novelty of the methology, this would improve the value of this manuscript.

The authors thank the reviewer for this comment. We are well aware of the small sample size. However, for logistical reasons, it was impossible to include more participants and we are currently unable to complete recruitment to meet the recommended target.

Nevertheless, several arguments can be advanced to support the publication of this work despite the small sample size:

-Firstly, Anderson and Vingrys have argued that "small samples may be sufficient to show the presence of an effect, but not to estimate the size of the effect. If the objective is only to show the existence of an effect, it is possible to avoid the cost of a large sample." In our study, the effect is quite marked, and in any case sufficient to obtain p-values <0.05.

This argument as well as bibliographic references has been added to the manuscript

Anderson AJ, Vingrys AJ. Small samples: does size matter? Invest Ophthalmol Vis Sci. 2001 Jun;42(7):1411-3. PMID: 11381039.

Indrayan A, Mishra A. The importance of small samples in medical research. J Postgrad Med. 2021 Oct-Dec;67(4):219-223. doi: 10.4103/jpgm.JPGM_230_21. PMID: 34845889; PMCID: PMC8706541.

- Secondly, we stressed several times in the manuscript that this was a pilot study, and we moderated the value of our results due to the small sample size.

-Third, a similar pilot study involving exactly the same small number of subjects with Autism Spectrum Disorders was accepted and published in the journal PlosOne (Lavenne, 2022) with a similar methodology, emphasizing that the small number of participants was not a sufficient obstacle to oppose publication.

- Moreover, no pathological features are reported (subtype of diagnosis, current pharmacological treatment).

We thank the reviewer for this comment. We have added data concerning the pathological characteristics of participants with AN (table 1) and specified that the participants were not taking any medication.

- I suggest to include other measures based on literature: for instance, body distortion might be associated with altered SOD.

The authors thank the reviewer for this comment. It would indeed be very interesting to include other measures based on the literature, for example concerning body distortion, which could be associated with alterations in SOD. However, this goes far beyond the scope of this preliminary work, which was to provide the first evidence of SOD alterations in anorexia nervosa and to characterize their type (egocentrism / heterocentrism). This proposal by the reviewer may, however, be the subject of future work.

- I would evaluate also visual abilities across groups (use of lens, glasses, other visual-corrective devices)

The authors would like to thank the evaluator for this comment. The participants' visual skills/abilities were indeed tested, since any visual deficit was considered an exclusion criterion for participants in both groups, which has been clarified in the manuscript.

*Discussion*

- Your results might be also related to multisensory integration deficits as highlighted by a recent systematic review. I suggest to include this aspect in the discussion section (for instance. Brizzi et al., 2023).

The reviewer's comment is quite pertinent. An entire paragraph of the discussion is already dedicated to the hypothesis that our results could be related to multisensory integration deficits. This has been made more obvious to the reader in the title of the dedicated paragraph and the suggested reference has been added (Brizzi et al., 2023).

- I suggest to extend the part related to egocentric-allocentric spatial frames (pag. 16-17)

The authors thank the reviewer for this comment. The section related to egocentric-allocentric spatial frames has been extended in the discussion.

- *“In particular, individuals with AN have been found to exhibit greater sensitivity to the rubber hand illusion (Botvinick & Cohen, 1998), indicative of a greater susceptibility to appropriating and embodying an object external to their own body, i.e., a more plastic bodily-self (Eshkevari et al., 2012)*” (pag. 18) – Actually, there are contrasted results concerning this aspect so I suggest a deeper discussion about contrasting results (for instance, Provenzano et al., 2019)

The authors thank the reviewer for this comment. It is true that the literature has highlighted contrasting results with regard to the sensitivity of AN patients to body illusion paradigms and this has been added in the manuscript. In particular, what the literature has highlighted is a stronger bodily illusion for body parts (notably the hands in the Rubber hand illusion) contrasting with similar levels of full-body illusion sensitivity in AN versus HC participants. A conceivable explanation for these contrasting results (rubber hand illusion versus full-body illusion) might reside in the body part where touch is delivered. In full-body illusions, touch is delivered to a highly salient and problematic part of the body for the AN, namely the area around the abdomen. Our results relating to a body part (in our case, the face) are therefore convergent with those reported in the literature for illusions limited to body parts in anorexia nervosa.

- “*These results may indicate that, in the AN individuals, the proprioceptive system would be less vulnerable to bias originating from visual information (i.e., AN individuals depend more heavily on proprioceptive information than on visual information when incongruent)*.” (pag. 19) – I would appreciate some evidence related to this aspect, since a recent systematic review highlithed interoceptive deficits in AN (see Malighetti et al., 2022)

The authors thank the reviewer for this request for clarification. It is worth clarifying the difference between proprioception and interoception. Indeed, proprioception refers to the sensation of knowing where one's limb/body is in space (Abboud et al., 2018) whereas interoception refers to sensations coming from the viscera (Ceunen et al., 2016). While deficits in interoceptive sensitivity have indeed been demonstrated in AN, studies that have focused on proprioception (at a unimodal level) suggest that proprioceptive processing is intact in people with anorexia nervosa.

With regard to multisensory integration (multimodal level), our results suggest that in patients with AN the proprioceptive system would be less vulnerable to visual information bias (i.e., people with anorexia rely more on proprioceptive information than visual information when it is incongruent)." These results are in line with previous work using the size-weight illusion (SWI), also known as the Charpentier illusion, which is a classic example of such a perceptual illusion. This illusion occurs when a smaller object is perceived as heavier than a larger object that has the same mass. Case showed a decrease in SWI in patients with AN, reflecting a decrease in the integration of visual and proprioceptive information, proprioceptive input being superior to visual input, consistent with our results.

Limitations

- What are other domains of SOD (pag. 23)?

As mentioned in the introduction, Self-other distinction (SOD) refers to the ability to distinguish one's own body, actions and mental representations from those of others . Distinguishing between self and other is therefore a unified process that spans several domains, in particular three domains that have been mainly studied, namely motor, cognitive and affective.

The task used in this study concerns perceptual SOD, i.e. the ability to identify one's own body (here, one's face) and distinguish it from others. Thus, it raises the question of whether the results of the present study can be generalized to the other SOD domains mentioned above.

- “*in the second part of the study, the postural and sensorimotor tasks were only applied to participants with AN. Therefore, the effects of these tasks in healthy subjects remained unknown*” (pag. 23) – I do not understand the reasons for this choice from a methodological point of view.

Our hypothesis was that patients with anorexia nervosa may exhibit sensorimotor deficits associated with lower levels of bodily self-consciousness as well as deficits in SOD. Our aim was to explore whether providing these subjects with external supports or stimuli (chosen in agreement with specialized rehabilitators) to receive bodily reinforcement could increase bodily self-consciousness and thus normalize SOD thresholds compared with healthy controls.

For this reason, we provided these supports only to participants with AN, while controls did not, in order to study this compensatory effect assuming that healthy control participants have intact bodily self-consciousness levels (without the need for supports).

This has been better explained in the manuscript, and this paragraph has been moved to the method section to justify this choice.

Interestingly, a recent article on the lived body in anorexia nervosa goes in the same direction as our findings. The authors indeed found that by allowing themselves to lean on the seat and back of the chair (during physiotherapy consultations), instead of perching on the edge of the chair, subjects suffering from anorexia nervosa felt more attentive and present in the here and now, indicating greater self-consciousness through postural supports (Naess 2022) . This reference has been added to the manuscript to support our hypothesis and results.

Naess CR, Kolnes LJ. A preliminary exploration of experiences of integrating the body in the self in two women with anorexia nervosa in view of phenomenological conceptualisations. J Eat Disord. 2022 Nov 1;10(1):153. doi: 10.1186/s40337-022-00675-x. PMID: 36320024; PMCID: PMC9628077.

In light of these considerations, I suggest authors to collect more data first of all. This would also improve the analysis. All other changes are minors and more related to a deeper analysis of the literature and/or style.

Once again, we'd like to thank the reviewer for his very constructive comments, and we're very hopeful that the answers and changes we've made will satisfy his requests for modifications.
