# Supplementary material for: Self /other recognition and distinction in adolescents with anorexia nervosa: A pilot study using a double mirror paradigm
Source: PLoS One. 2025 Jan 2;20(1):e0309548. doi: 10.1371/journal.pone.0309548 (PMC11695000; doi:10.1371/journal.pone.0309548)
Supplement: Attachment — Submitted filename: Response to reviewers 2 .docx [file pone.0309548.s005.docx]

**Response to reviewers**

Reviewer #1: The authors have addressed all items that were identified as issues in the first iteration of the draft. I think this piece is ready for publication.

The authors would like to thank the reviewer for his comments and advice on the first draft, which helped to improve the article. They are also very grateful for his support and interest in our work.

Reviewer #3: I really like the idea behind this paper, the innovative approach, and the findings are of interest for a broader audience.
I have some suggestions to improve the work and some questions.

First of all, the authors would like to thank the reviewer for his positive comments, for the interest and time he devoted to their work and, above all, for his in-depth knowledge of the issue, which enabled them to significantly improve the article. They hope that the response to his comments and the changes they have made will confirm that this work is worthy of publication.

- Please add "pilot study" to the title and include the n in the abstract

The authors would like to thank the reviewer for this comment. The qualifier "pilot" has been added to the title and the n included in the abstract.

- You indicate that all the data is available, but this does not seem to be the case. I do not find any raw data in the supplement.

The authors would like to thank the reviewer for this comment. Raw data have been added as a supplement

- You give a very nice overview of the existing literature, except you are missing a whole large field of studies that are very important with regard to body perception, self-other-distinction, and sensorimotor integration. There are many studies on the perception and processing of social, affective touch, which has been suggested to play a crucial role in the development of the bodily self and might specifically altered in anorexia. These findings should be integrated both in the introduction and in the discussion

Here are some references:

Crucianelli, L., Cardi, V., Treasure, J., Jenkinson, P. M., & Fotopoulou, A. (2016). The perception of affective touch in anorexia nervosa. Psychiatry research, 239, 72-78.Frost-Karlsson, M., Capusan, A. J., Perini, I., Olausson, H., Zetterqvist, M., Gustafsson, P. A., & Boehme, R. (2022). Neural processing of self-touch and other-touch in anorexia nervosa and autism spectrum condition. NeuroImage: Clinical, 36, 103264.
Davidovic, M., Karjalainen, L., Starck, G., Wentz, E., Björnsdotter, M., & Olausson, H. (2018). Abnormal brain processing of gentle touch in anorexia nervosa. Psychiatry Research: Neuroimaging, 281, 53-60.
Bischoff-Grethe, A., Wierenga, C. E., Berner, L. A., Simmons, A. N., Bailer, U., Paulus, M. P., & Kaye, W. H. (2018). Neural hypersensitivity to pleasant touch in women remitted from anorexia nervosa. Translational psychiatry, 8(1), 161.

The authors would like to thank the reviewer for this very pertinent commentary and additional bibliography. We have integrated the literature about alterations in social and affective touch in anorexia, emphasizing its crucial role in the development of the bodily self and in the self/other distinction, both in the introduction and in the discussion with two additional paragraphs added on pages 6 and 23 respectively. All the suggested bibliography, and even more, has been added.

- Do you have the BMI values for the controls?

BMI values for the controls were not analyzed. However, it was checked at inclusion that controls had a normal BMI in addition to the absence of eating disorders. This was specified in the methodology section (page 9).

- Did you colllect Autism quotient scores?

We thank the reviewer for this question. We did not collect participants' autistic quotient scores, but the reviewer is right to point out that this would have been relevant.

Indeed, there is an overlap in symptomatology between ASD and anorexia nervosa. Moreover, in a previous study using the Alter ego double mirror system, we showed alterations in the self/other distinction in participants with ASD compared with controls.

However, the aim of this work was above all to demonstrate for the first time the existence of SOD in AN. Consequently, the question of comparing these two populations was beyond the scope of this pilot study, but deserves to be explored in a future study. This has been added to the manuscript (page 26).

- I find the description of the tasks somewhat confusing, i.e. it is unclear how often the back and forth passage occurs. Maybe you could start with a simple overview over the procedure (how often is each condition repeated). This is also unclear for the sensorimotor stimulations. Were they all just presented once? Is it always a back and forth neutral followed by one of the sensorimotor conditions?

We thank the reviewer for this comment.

We have improved the description of the procedure by starting with a simple overview at the beginning of the "protocol and task" section (page 11) as below:

The experimental procedure had a duration of approximately 90 minutes and was divided into 2 parts:

- First, the *neutral condition*, lasted 15 minutes and consisted of a « back-and-forth » passage, i.e. TDCs moved progressively from self to other, then from other to self while, during the same period of time, participants with AN experienced the opposite condition, i.e. they moved from other to self, then from self to other ;

-The second part, called the *sensorimotor stimulation condition*, lasted 60 minutes and involved exposing participants to the same light intensity conditions as described in the neutral condition, i.e. a "back-and-forth » passage, but repeated 5 times with a different stimulus applied to the participants with AN during each passage.

Thus, the whole task consisted of 6 back-and-forth passages: one in neutral condition, then 5 with different successive sensory stimuli only for AN participants.

There was a 10-min pause between the first and second parts to allow attentional recovery.

- How long did each trial last? How long was the complete experiment?

We thank the reviewer for this comment. The duration of each test has been specified in the manuscript as indicated in the new paragraph above (Page 11).

- Were participants allowed to move / grimace during the tasK? What was the instruction?

We thank the reviewer for this comment. It was specified in the methodology section that participants were positioned so as to allow strict alignment of the eyes of both partners in the mirror and that they were asked to maintain this alignment throughout the task, without making any body or facial movements (including no grimace). Moreover, the instruction given to participants was clarified (page 11).

- Do I see this correctly, that you do not correct for multiple comparisons?

The reviewer is right, we made no multiple comparisons adjustments. Indeed, adjustments for making multiple comparisons in large bodies of data are recommended to avoid rejecting the null hypothesis too readily. Howewer, a policy of not making adjustments for multiple comparisons is sometimes preferable because it will lead to fewer errors of interpretation when the data under evaluation are not random numbers but actual observations on nature. (Rothman, 1990, Barnett et al., 2022).

Rothman, K.J. (1990). No adjustments are needed for multiple comparisons .Epidemiology, 1: 43-46.

Barnett, M. J., Doroudgar, S., Khosraviani, V., & Ip, E. J. (2022). Multiple comparisons: To compare or not to compare, that is the question. *Research in social & administrative pharmacy : RSAP*, *18*(2), 2331–2334. https://doi.org/10.1016/j.sapharm.2021.07.006

- An alternative explanation could be that the AN participants are worse at matching their actions with the sensory input, i.e. they have problems with predicting the sensory outcome of their own action (so they do not identify when the mirror image does not match their movement - even if they kept their face still, there is still movement due to blinking the eyes, breathing etc).

The authors thank the reviewer for this comment. This additional hypothesis has been added to the discussion (page 23).

- Another alternative that I do not see can be controlled for is: I assume anorexia patients do not like seeing their own face. This could mean a) they do not often look in the mirror and are simply not used to seeing their own face (however, then they would probably tend to identify the other faster), or b) they really dislike seeing their own face and are therefore more sensitive to perceiving it.

We thank the reviewer for this comment. We believe that our results can not be explained by an attentional or emotional bias related to the fact that subjects with AN dislike seing their own face in the mirror (i.e. they would have avoided or be more sensitive to seeing it ). Indeed, if this hypothesis were correct, this tendency should have been the same regardless of the direction of morphing.

The answer to this question is now included in the "limitation" section of our results, when we state that our results cannot be explained by differences in attentional performance or information processing (page 27).

- The sensorimotor feedback from the back and foot support could also simply be a distraction from the over-focus on the own face?

We thank the author for this comment.

We believe that the answer we gave to the previous question also applies here: if the hypothesis was that of distraction due to excessive focus on one's own face, this effect should have been found similarly regardless of the direction of morphing.

Moreover, if the distraction hypothesis raised by the reviewer were correct, we should have expected an even greater distraction effect for the most unusual sensorimotor tasks (such as sitting on an unstable cushion). However, it is the effect of the task reinforcing the median axis of the body (which is not the most unusual one) that stands out significantly in our results, and confirms the clinical hypothesis of body therapists specialized in anorexia, even if our results still need to be replicated on a larger sample.

This has been added to the manuscript (page 28).

Minor:

- introduction: third line, should be "a growing number of studies"

The authors thank the reviewer for this remark. The wording has been improved.

- I am also not a native English speaker, so pardon me if this is incorrect, but I find the formulation "AN individuals were installed in the BFR condition" very strange.

The authors thank the reviewer for this remark. The wording has been improved (pages 17, 18 and 23).
